# Cytogenomics of *Tetragonisca angustula* Reveal Unique and Shared satDNA Families Within *Tetragonisca* and Closely Related Genera (Apidae: Meliponini)

**DOI:** 10.3390/genes17101254

**Published:** 2026-10-10

**Authors:** Marina Souza Cunha, Gisele Amaro Teixeira, Lucio Antonio de Oliveira Campos, Denilce Meneses Lopes

**Affiliations:** 1 Instituto de Veterinária, Universidade Federal Rural do Rio de Janeiro, Campus Seropédica, Seropédica CEP 23891-970, RJ, Brazil; 2 Laboratório de Citogenética de Insetos, Departamento de Biologia Geral, Universidade Federal de Viçosa, Campus Viçosa, Viçosa CEP 36570-900, MG, Brazil; g.amaroteixeira@gmail.com (G.A.T.); lucioaoc@gmail.com (L.A.d.O.C.)

**Keywords:** chromosome evolution, heterochromatin composition, jataí, repetitive DNA, stingless bees

## Abstract

Background/Objectives: Only a handful of stingless bees have had their satDNAs characterized using a cytogenomic approach, highlighting the importance of these repetitive sequences as major heterochromatin constituents. Recently, the genome of *Tetragonisca angustula* became available, providing an opportunity to investigate the diversity of satDNAs in this species with its remarkably large genome size. Our goal was to identify satDNAs in the genome of *T. angustula* and investigate their chromosomal distribution in populations of *T. angustula* and *Tetragonisca fiebrigi* from different geographical locations, as well as in the closely related species *Duckeola guilianii* and *Frieseomelitta varia* and other more distantly related species. Additionally, we also investigated the variability in rDNA clusters among different *T. angustula* and *T. fiebrigi* populations. Methods: Genomic analyses were performed using RepeatExplorer2 and TAREAN, and chromosomal investigations were conducted using fluorescent *in situ* hybridization using satDNAs and rDNA sequences as probes. Results: Four satDNA families were identified, accounting for 56.405% of the *T. angustula* analyzed genome fraction. Among these, TangSat01-304 is the most abundant, constituting the main heterochromatin constituent of both *Tetragonisca* species. TangSat02-316 is present on the centromeres of both *Tetragonisca* species, being shared with several other Meliponini species. TangSat03-129 colocalizes with rDNA genes, being more conserved in the number of sites in *T. angustula* and more variable in *T. fiebrigi*, including markings on its B chromosomes. This satDNA is also shared with other stingless bee species. Finally, TangSat04-229 revealed a single euchromatic cluster on one chromosome pair in both *Tetragonisca* species, being exclusive to this genus, like TangSat01-304. Conclusions: Our results demonstrated that the genome size expansion in *Tetragonisca* was largely driven by the accumulation of satDNAs, particularly TangSat01-304.

## 1. Introduction

Satellite DNAs (satDNAs) consist of sequences tandemly repeated many times, whose total length may reach several hundreds of base pairs (reviewed in [1,2]). This kind of element is present mainly in the heterochromatin, possibly participating in its formation [3]. Although mostly unknown, some roles of satDNAs have been suggested in centromere functionality or in its correct segregation during the cell cycle (reviewed in [4]). As some satellites can be differentially transcribed in specific tissues or developmental stages, a potential function during development has also been suggested [1,5,6].

The library hypothesis proposes that phylogenetically related species may share an ancestral collection of satDNAs inherited from a common ancestor, which may undergo changes following speciation [2,7]. Therefore, satDNAs can be conserved over long evolutionary times, or, alternatively, they can experience stochastic changes and become species-specific or specific to closely related groups. For example, the most abundant satDNA from the *F. varia* (Lepeletier, 1836) genome (FvarSat01-306) is shared with other stingless bee genera dating back at least 44 Mya [8]. On the other hand, the most abundant satellite ThyaSat01-301 from the stingless bee *Trigona hyalinata* (Lepeletier, 1836) is shared only among *Trigona* species from clade B but not among *Trigona* species from clade A [9]. The sister clades of *Trigona* diverged around 19 Mya [10].

SatDNA enrichment can vary greatly among species, from being small constituents of the genome to becoming major components of the repeatome [1]. For example, these elements compose 2.84% of the genome of *Melipona (Melipona) quadrifasciata* Lepeletier, 1836, whereas it represents 38.41% of the genome of *Melipona (Michmelia) scutellaris* Latreille, 1811 [11]. These two *Melipona* species share most satDNA families in their genomes, but they vary largely mainly due to the amplification of unique repetitions, such as MquaSat01-586 in the former species and MscuSat01-195 in the latter [11]. In this sense, some satellites are shared among different *Melipona* subgenera, but the most abundant sequence is unique to each one [11,12,13].

According to the available molecular phylogenies of the Meliponini tribe (Apidae), the genera *Frieseomelitta*, *Duckeola*, and *Tetragonisca* belong to the same branch [10,14]. Studies on the genus *Tetragonisca* have shown the sharing of a repetitive sequence on the whole heterochromatic portion of *T. angustula* (Latreille, 1811) and *T. fiebrigi* (Schwarz, 1938) chromosomes, including *T. fiebrigi* B chromosomes, but not with other Meliponini species [15,16]. Additionally, Vignati et al. [8] showed that the most abundant satDNA of *F. varia*, FvarSat01-306, is present in the centromeric region of *T. angustula* and *D. guilianii* (Spinola, 1853) chromosomes.

Other constituents of the repeatome are ribosomal genes (rDNA), which consist of tandemly repeating units of different DNA sequences (genes and spacers) [17]. The number of repeat units of rDNA varies greatly among eukaryotes, from 39 to 19,300 in animals [18]. Only one pair of rDNA clusters is supposed to be the ancestral condition in the Neotropical Meliponini clade, independently increasing in several genera, including *Tetragonisca* [16,19]. The terminal position of the ribosomal clusters may facilitate their dispersion to different chromosomes within the karyotype through mechanisms such as translocations, unequal exchange, and ectopic recombination during meiosis [20,21,22].

Massive heterochromatin amplification is reported to have occurred during the evolution of some Meliponini stingless bees [23]. For example, the genus *Tetragonisca* has almost double the genome size (1C = 0.9 pg) compared to closely related genera *Duckeola* (1C = 0.5 pg) and *Frieseomellita* (1C = 0.5 pg) [23,24]. The genomic expansion of repetitive sequences, such as satDNAs, rDNAs, and transposable elements, could account for this difference. 

Recently, the genome sequence of *T. angustula* became available [25], presenting an opportunity to investigate the presence of different repetitive DNA sequences. The goal of this study was to identify satDNA families present in the available genome of *T. angustula* and investigate their chromosomal distribution in different populations of *T. angustula* and *T. fiebrigi*, as well as in the closely related species *D. guilianii* and *F. varia* and other more distantly related species. Together, these analyses provide a framework to explore the evolutionary dynamics of repetitive DNA and their contribution to heterochromatin differentiation in the genus *Tetragonisca*.

## 2. Material and Methods

### 2.1. Biological Material

Individuals of many *T. angustula* and *T. fiebrigi* populations were sampled from different Brazilian regions (Table 1; Figure S1). Species identification followed [26,27]. Collecting permit number SISBIO-ICMBio 80435-5 was issued to Marina Souza Cunha. Adult individuals were fixed in absolute ethanol and stored at −20 °C at the Laboratório de Citogenética de Insetos of the Universidade Federal de Viçosa, Minas Gerais, Brazil. Voucher specimens were deposited in the Coleção Entomológica “Prof. J.M.F. Camargo”, at Universidade de São Paulo, Ribeirão Preto, São Paulo, Brazil (voucher numbers RPSP4322–RPSP4336, RPSP5157).

### 2.2. SatDNA Analysis in the Genome of T. angustula

The raw genome data of *T. angustula* was obtained from the publicly accessible database Sequence Read Archive (SRA)—NCBI, with the accession number SRX22081749 [25]. The sequencing data comprises 27.6 Gb [25], corresponding to a high genome coverage of approximately 30×, based on its genome size of 929 Mb [28]. satDNAs were identified using pipelines RepeatExplorer2 and TAREAN, available on the Galaxy platform (Galaxy Version 2.3.8.1) (accessed in February 2025). These analyses followed the protocols described by Novák et al. [29,30]. The pre-processing of paired-end reads was performed using default settings, which included the quality filtering of the reads, discarding the single reads and keeping complete pairs, and the interlacing of two FASTQ files. RepeatExplorer2 clustering and TAREAN were run with default parameters using a random sample of three million reads, which corresponded to 0.3× coverage of the genome size (1C = 0.95 pg or 929 Mb; see [28]).

We compared the identified satDNA sequences to check for a possible identity between them using Geneious software V4.8 [31]. Considering their level of sequence identity, satDNA sequences were classified into families (>80%) and named according to Ruiz-Ruano et al. [32]. These sequences were deposited in GenBank with accession numbers: PZ803731, PZ803732, PZ803733, and PZ803734. Also, satDNA sequences were compared to the recognition site of the restriction enzyme Bsp 68I used by Pereira et al. [15] which yielded a heterochromatic probe for *T. angustula* and *T. fiebrigi* chromosomes. In addition, all satDNA sequences in this study were compared with sequences previously deposited on Repbase v20.10 [33] and in the GenBank/NCBI DNA databases with the BLASTn tool [34] to identify possible similarities.

The abundance and divergence of each satDNA family were estimated by means of RepeatMasker (Version 4.1.1) [35] using the RMBlast search engine. A total of ten million randomly selected reads were used for this analysis. Satellite abundance was estimated based on its proportion in the genome, and the sum of mapped nucleotides belonging to one specific satDNA was divided by the total number of nucleotides in the library. Satellite divergence was determined using the Kimura 2-parameter (K2P) using the calcDivergenceFromAlign.pl script in RepeatMasker software [35]. Repeat landscapes were generated by comparing the sequence divergence versus the abundance of each satDNA family.

### 2.3. Chromosomal Mapping of SatDNAs and 18S rDNA Region

Mitotic chromosomes from the stingless bee species (Table 1) were obtained from the cerebral ganglia of larvae [36]. The physical mapping of satDNA sequences was performed in *T. angustula* and phylogenetically close species, i.e., *T. fiebrigi*, *D. guilianii*, and *F. varia*, as well as in other Meliponini stingless bees, i.e., *F. schrottkyi* (Friese, 1900), *P. phrynostoma* Moure, 2004, *N. testaceicornis* (Lepeletier, 1836), *S. xanthotricha* Moure, 1950, *P. helleri* (Friese, 1900), *Melipona asilvai* Moure, 1971, *Melipona quadrifasciata* Lepeletier, 1836, and *L. muelleri* (Friese, 1900).

We produced probes for the four satDNAs found in the genome of *T. angustula* via a Polymerase Chain Reaction (PCR) using the following manually designed specific primers: TangSat01-304 F- 5′ AATAAATTCCGACGCAATGGG 3′ and R- 5′ AGGGAACAGTCGCATTTTATC 3′; TangSat02-316 F- 5′ CTACTTAGATAGATGCAGACG 3′ and R- 5′ CGTCTGCATCTATCTAAGTAG 3′; TangSat03-129 F- 5′ CTCGGCCAGTCGTGTCAC 3′ and R- 5′ CCTCGAGTCTCGTTCCGG 3′; and TangSat04-229 F- 5′ GAAATTGTAAAGAGACTTTATGG 3′ and R- 5′ AAACATTTATTACAAAGGCAAGG 3′. The genomic DNA of *T. angustula* was extracted using the DNeasy Kit (Qiagen Inc., Redwood City, CA, USA), following the manufacturer’s instructions. The PCR solutions totaling 10 μL contained 5.52 μL of H_2_O, 1 μL of template DNA (100 ng/μl), 0.4 μL of each primer (10 mmol/L) (forward and reverse), 2 μL of 5× Color-less GoTaq Flexi buffer, 0.08 μL of dNTPs (20 mmol/L), 0.4 μL of MgCl2 (25 mmol/L), and 0.2 μL of GoTaq Flexi DNA Polymerase (5 U/μL) (Promega, Maddison, WI, USA). PCR conditions included an initial denaturation step at 94 °C for 5 min and 30 cycles at 94 °C (30 s), 60 °C (30 s), and 72 °C (80 s), plus a final extension at 72 °C for 5 min. The PCR products were checked on a 1% agarose gel.

The mapping of the four satDNA probes was performed using the fluorescence in situ hybridization (FISH) technique [37]. Probes were labeled by an indirect method with digoxigenin-11-dUTP using the Dig-Nick-Translation Mix kit (Roche Applied Science, Mannheim, Germany). The identification of probe signals was performed using antidigoxigenin–rhodamine (Roche Applied Science, Mannheim, Germany). Chromosomes were counterstained with 4′,6-Diamidino-2-Phenylindole (DAPI) (Fluoroshield with DAPI, Sigma-Aldrich, Darmstadt, Germany).

Additionally, we mapped the 18S rDNA gene in the chromosomes of different colonies of *T. angustula* and *T. fiebrigi*. The 18S rDNA probe was obtained through PCR using the following primers: F- 5′GTCATATGCTTGTCTCAAAGA 3′ and R- 3′TCTAATTTTTTCAAAGTAAACGC 5′. This probe was labeled and the signal detected as indicated previously for the satDNAs, and the chromosomes were also counterstained with DAPI. Metaphases were captured on an Olympus BX60 microscope equipped with an Olympus DP23M camera (Olympus, Tokyo, Japan) using CellSens imaging software (Standard Version 4.2).

## 3. Results

Bioinformatic analysis identified four satDNA families in the genome of *T. angustula*: TangSat01-304 represents 55.705% of the genome, with a monomer length of 304 base pairs (bp); TangSat02-316 represents 0.435% of the genome, with a monomer length of 316 bp; TangSat03-129 represents 0.211% of the genome, with a monomer length of 129 bp; and TangSat04-229 represents 0.054% of the genome, with a monomer length of 229 bp. Notably, the most abundant satellite, TangSat01-304, was recovered only using the TAREAN pipeline, whereas TangSat03-129 was detected exclusively by RepeatExplorer2.

**Figure 1:**
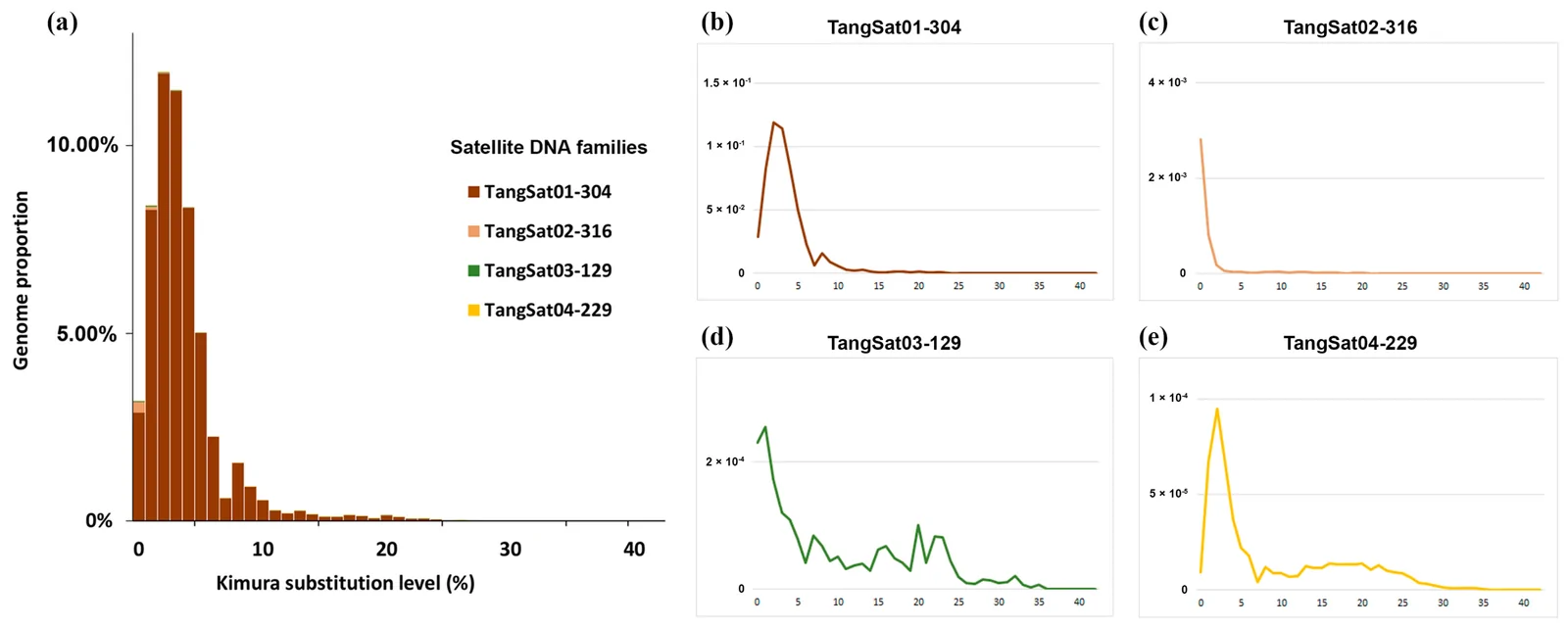
(**a**) The general landscape for all satDNAs identified on the genome of *T. angustula*, evidencing the predominance of TangSat01-304. (**b**,**c**) Landscapes for TangSat01-304 and TangSat02-316 showing an abundance peak at low divergence values (about 5%), revealing its recent amplification and high sequence homogenization. (**d**,**e**) Landscapes for TangSat03-129 and TangSat04-229 showing an abundance peak at high divergence values, indicating the accumulation of mutations in these sequences.

Together, the four satellite families showed a total abundance of 56.405% of the *T. angustula* genome, considering the analyzed genomic fraction. The satellites TangSat01-304, TangSat02-316, and TangSat04-229 showed a predominance of AT base pairs, with 63.5%, 63.9%, and 62.4%, respectively, whereas TangSat03-129 exhibited a higher GC content with 62.8%. The mean divergence of the satDNAs was 3.95%, 1.73%, 9.35%, and 10.14% for TangSat01-304, TangSat02-316, TangSat03-129, and TangSat04-229, respectively. According to the landscape graph (abundance versus divergence), two satDNA families, including the most abundant TangSat01-304, showed an abundance peak at low divergence (<5%) (Figure 1a–c). However, the other two satDNA families showed an abundance peak at higher divergence values (>5%) (Figure 1d,e).

**Figure 2:**
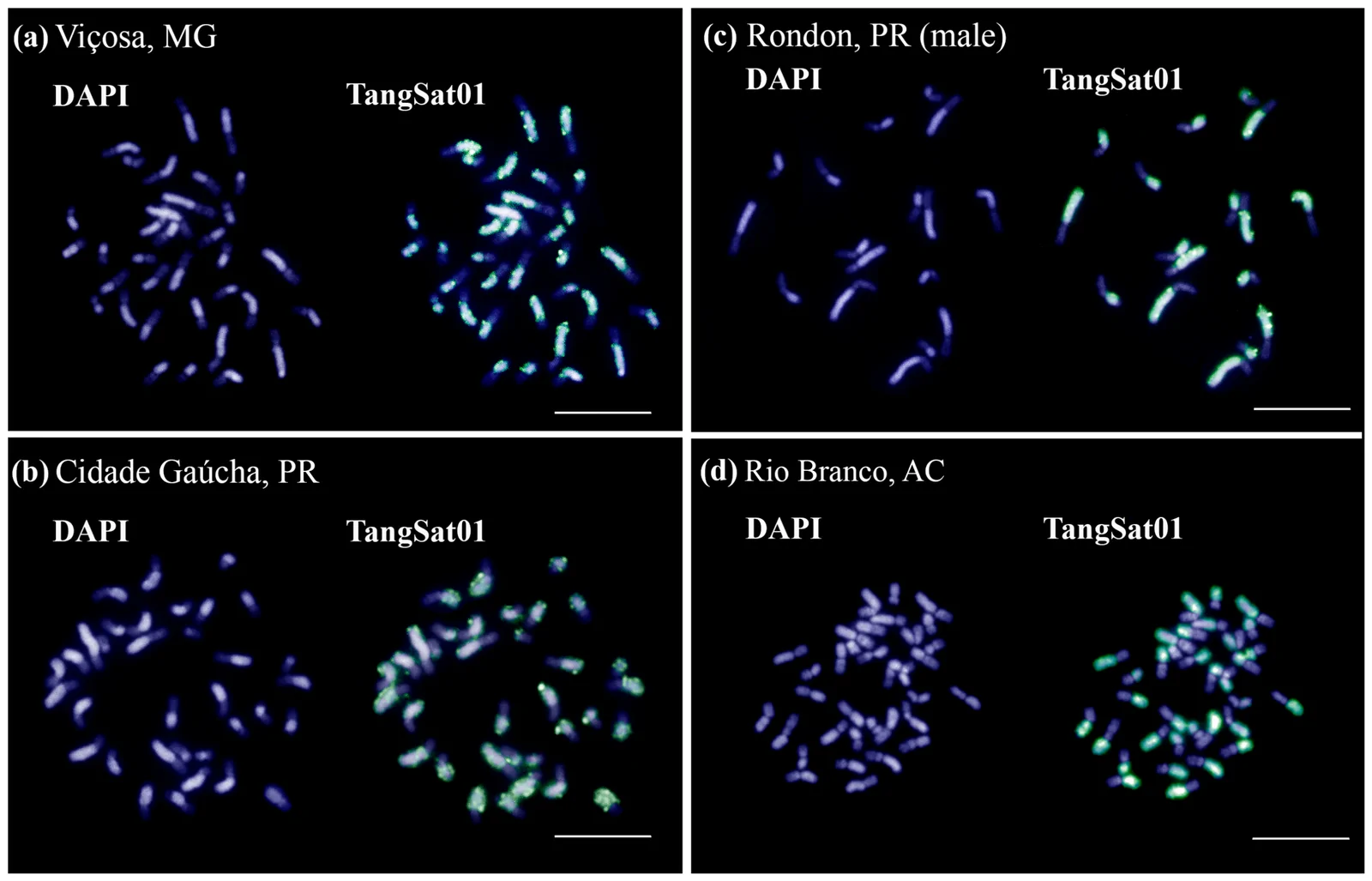
The chromosomal distribution of TangSat01-304 in the karyotypes of *T. angustula* from: (**a**) Viçosa, MG; (**b**) Cidade Gaúcha, PR; (**c**) Rondon, PR; (**d**) Rio Branco, AC. Chromosomes are shown stained with DAPI (blue) to highlight heterochromatic regions and merged with the satDNA probe (green). Scale bars = 10 µm.

**Figure 3:**
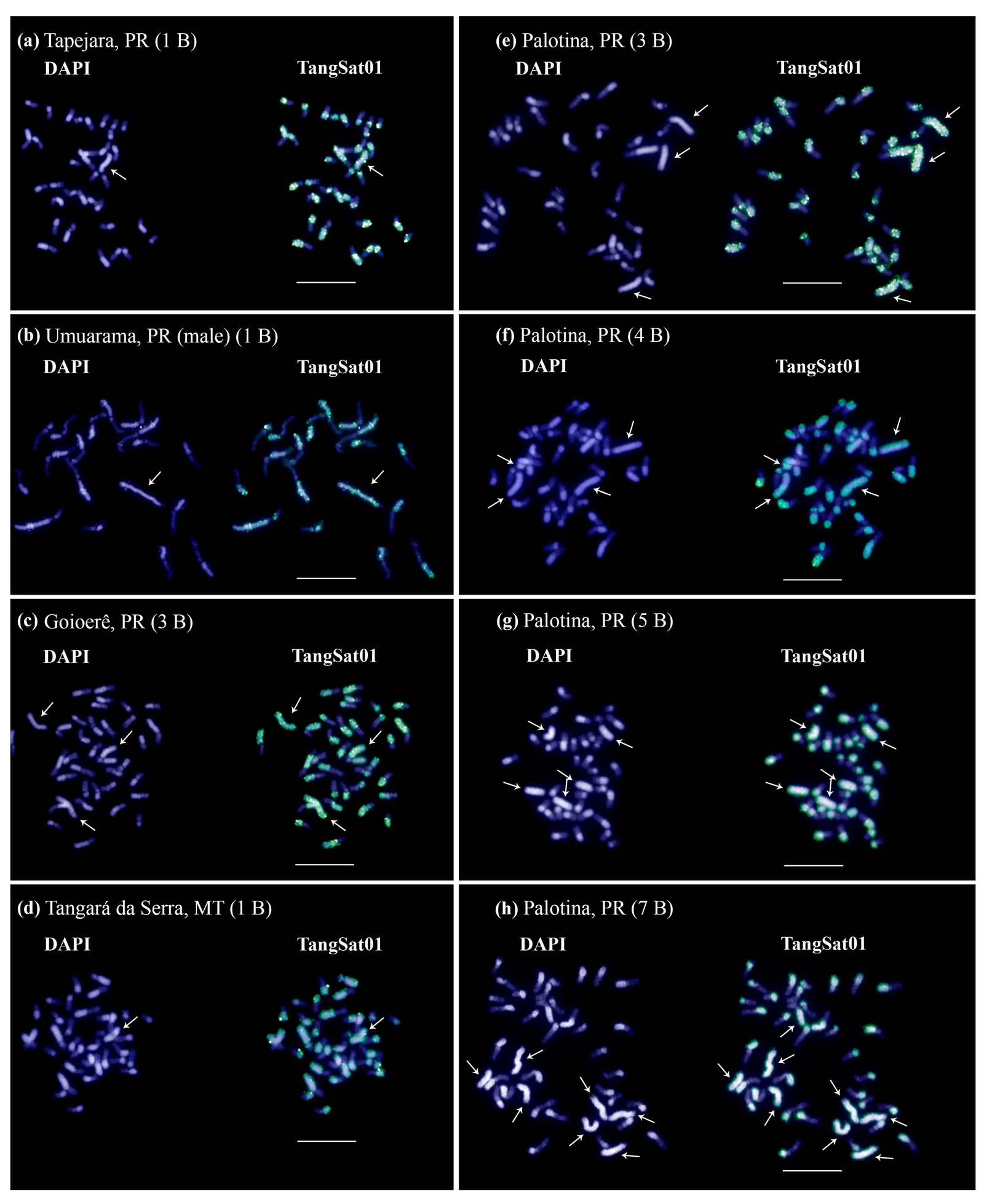
The chromosomal distribution of TangSat01-304 in the karyotypes of *T. fiebrigi* from: (**a**) Tapejara, PR; (**b**) Umuarama, PR; (**c**) Goioerê, PR; (**d**) Tangará da Serra, MT; (**e**–**h**) Palotina, PR. Chromosomes are shown stained with DAPI (blue) to highlight heterochromatic regions and merged with the satDNA probe (green). Arrows indicate the B chromosomes. Scale bars = 10 µm.

FISH assays with the TangSat01-304 probe revealed hybridization signals comprising the heterochromatic portions of all chromosomes of *T. angustula* and *T. fiebrigi*, including the B chromosomes exclusive of the latter species from different locations (Figure 2 and Figure 3). This satDNA family was not present in the chromosomes of *D. guilianii* or *F. varia* (Table 1). The digestion of *T. angustula* genomic DNA with Bsp68I yielded a band pattern in a ladder with a cleavage profile in repetitive regions, and FISH analysis revealed that this repetitive DNA was located on one arm of all chromosomes [15]. This repetitive DNA likely corresponds to TangSat01-304, which harbors a restriction site for this enzyme at positions 291–296 in its consensus sequence (Figure S2). Furthermore, both repetitive DNAs displayed the same hybridization pattern.

**Figure 4:**
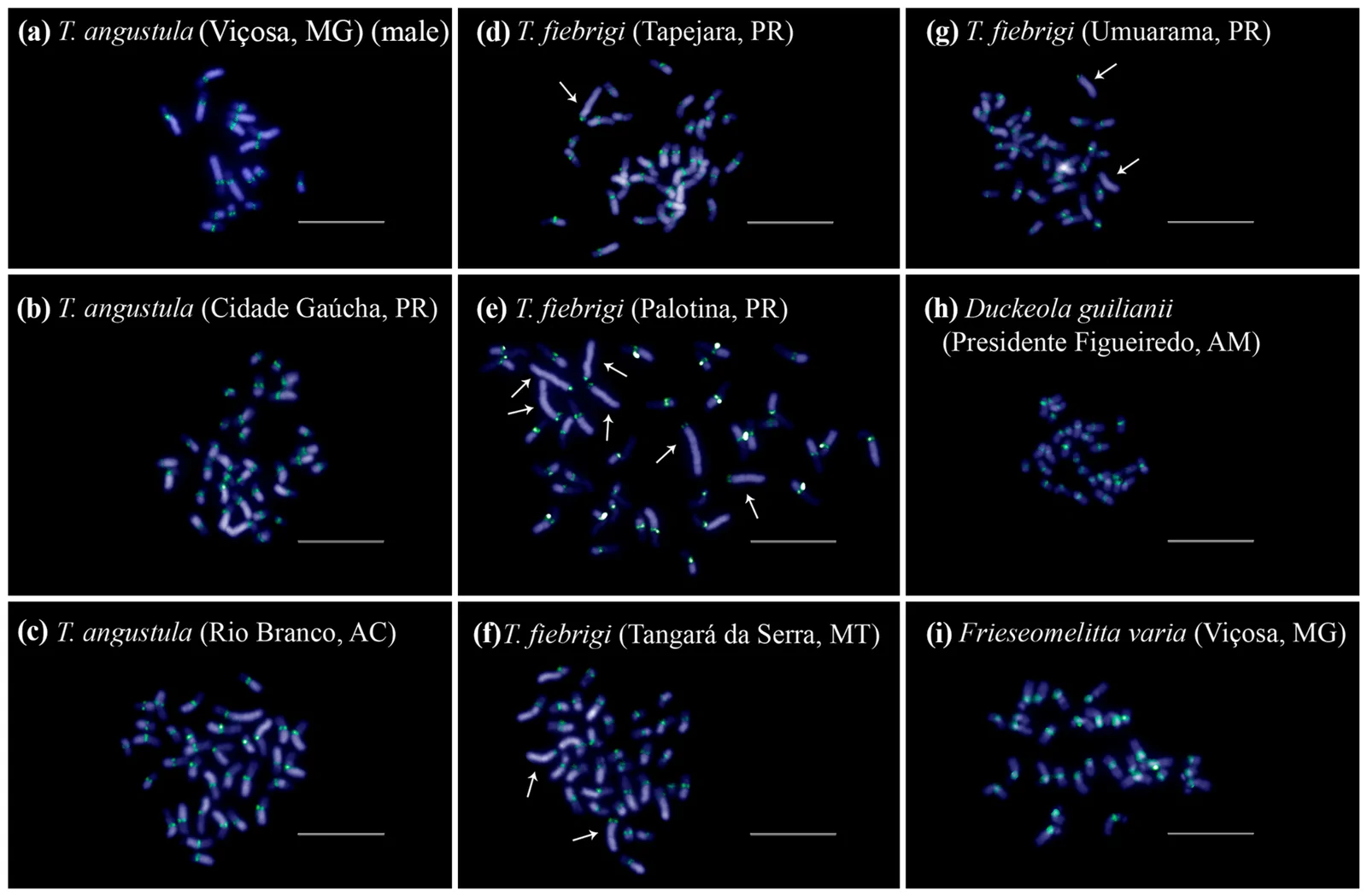
The chromosomal distribution of TangSat02-316 in the karyotypes of *T. angustula* from (**a**) Viçosa, MG; (**b**) Cidade Gaúcha, PR; and (**c**) Rio Branco, AC, as well as *T. fiebrigi* from (**d**) Tapejara, PR; (**e**) Palotina, PR; (**f**) Tangará da Serra, MT; (**g**) Umuarama, PR; and (**h**) *D. guilianii* from Presidente Figueiredo, AM, and (**i**) *F. varia* from Viçosa, MG. Chromosomes are stained with DAPI (blue), and the satDNA signals are shown in green. Arrows indicate the B chromosomes. Scale bars = 10 µm.

FISH assays with the TangSat02-316 probe revealed hybridization signals in the centromeric region of *T. angustula* and *T. fiebrigi* chromosomes and in the euchromatic extremity of the B chromosomes (Figure 4a–g). This probe also marked the centromeric regions of *D. guilianii* chromosomes and the majority of *F. varia* heterochromatin (Figure 4h,i). This satellite showed 80% similarity with FvarSat01-306 from *F. varia*, indicating that they belong to the same satDNA family (Figure S3).

**Figure 5:**
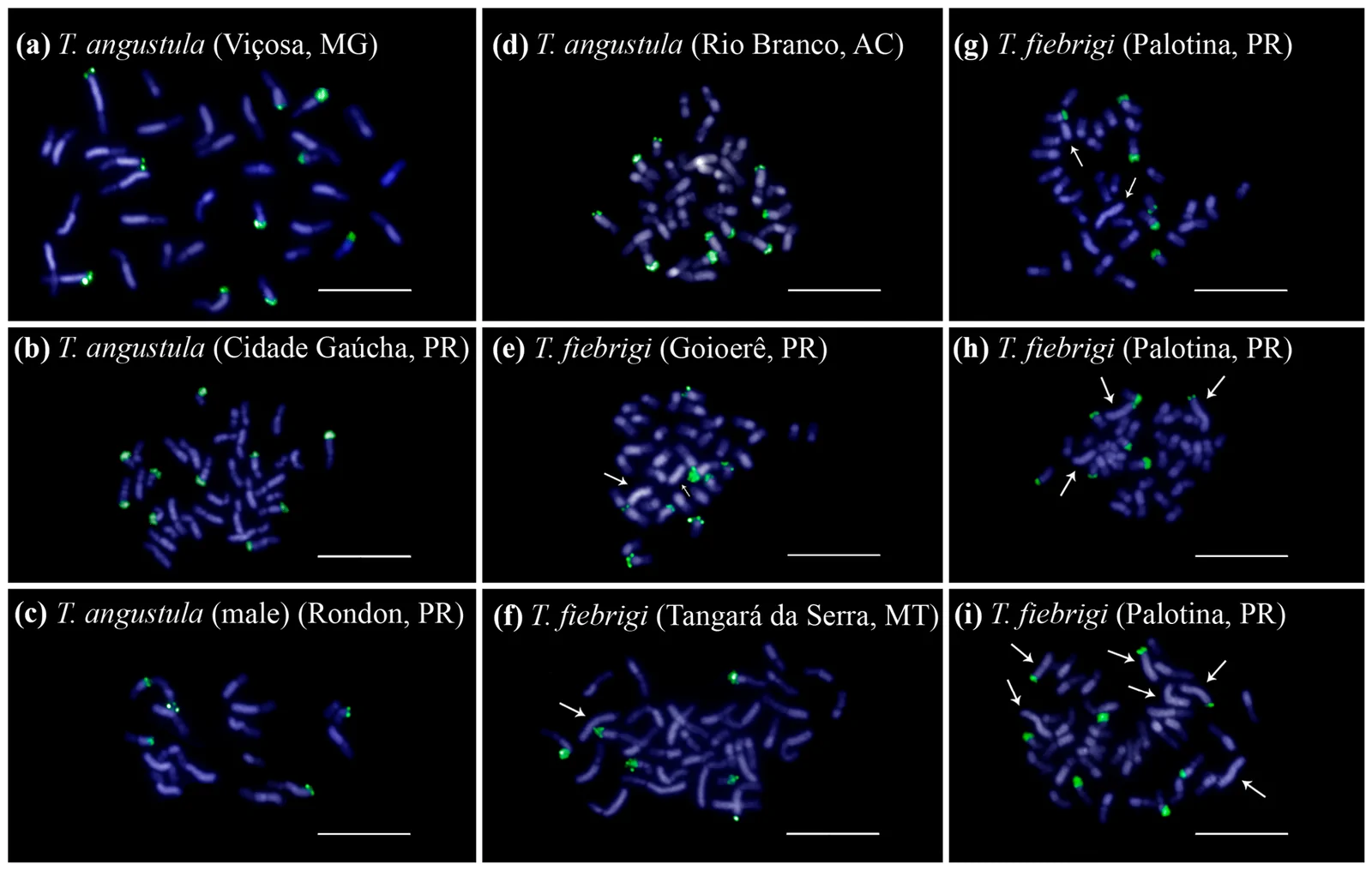
The chromosomal distribution of TangSat03-129 in the karyotypes of *T. angustula* from (**a**) Viçosa, MG; (**b**) Cidade Gaúcha, PR; (**c**) Rondon, PR; and (**d**) Rio Branco, AC, as well as *T. fiebrigi* from (**e**) Goioerê, PR; (**f**) Tangará da Serra, MT; and (**g**–**i**) Palotina, PR. Chromosomes are stained with DAPI (blue), and the satDNA signals are shown in green. Arrows indicate the B chromosomes. Scale bars = 10 µm.

**Figure 6:**
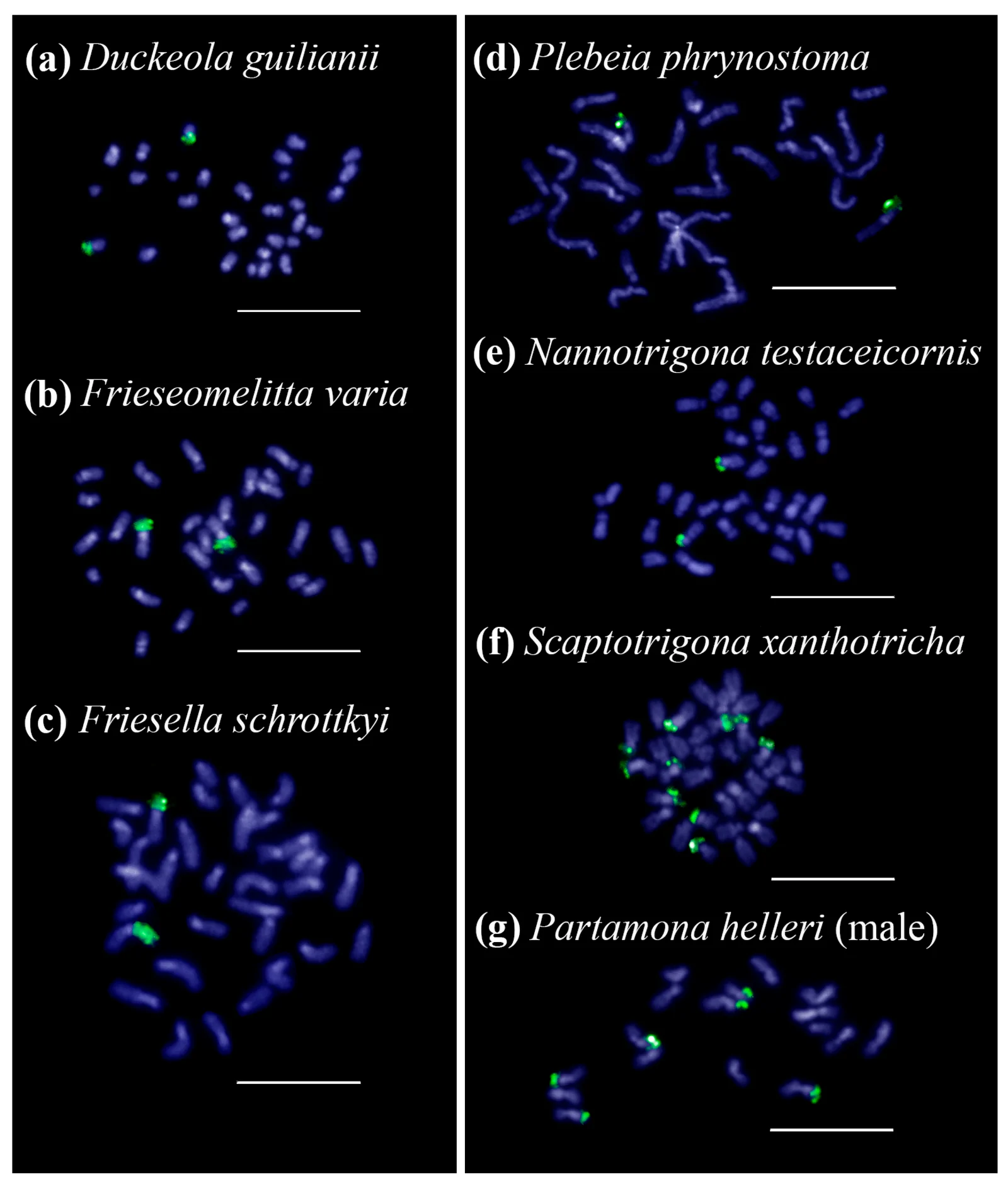
The chromosomal distribution of TangSat03-129 in the karyotypes of (**a**) *D. guilianii*; (**b**) *F. varia*; (**c**) *F. schrottkyi*; (**d**) *P. phrynostoma*; (**e**) *N. testaceicornis*; (**f**) *S. xanthotricha*; and (**g**) *P. helleri*. Chromosomes are stained with DAPI (blue), and the satDNA signals are shown in green. Scale bars = 10 µm.

**Figure 7:**
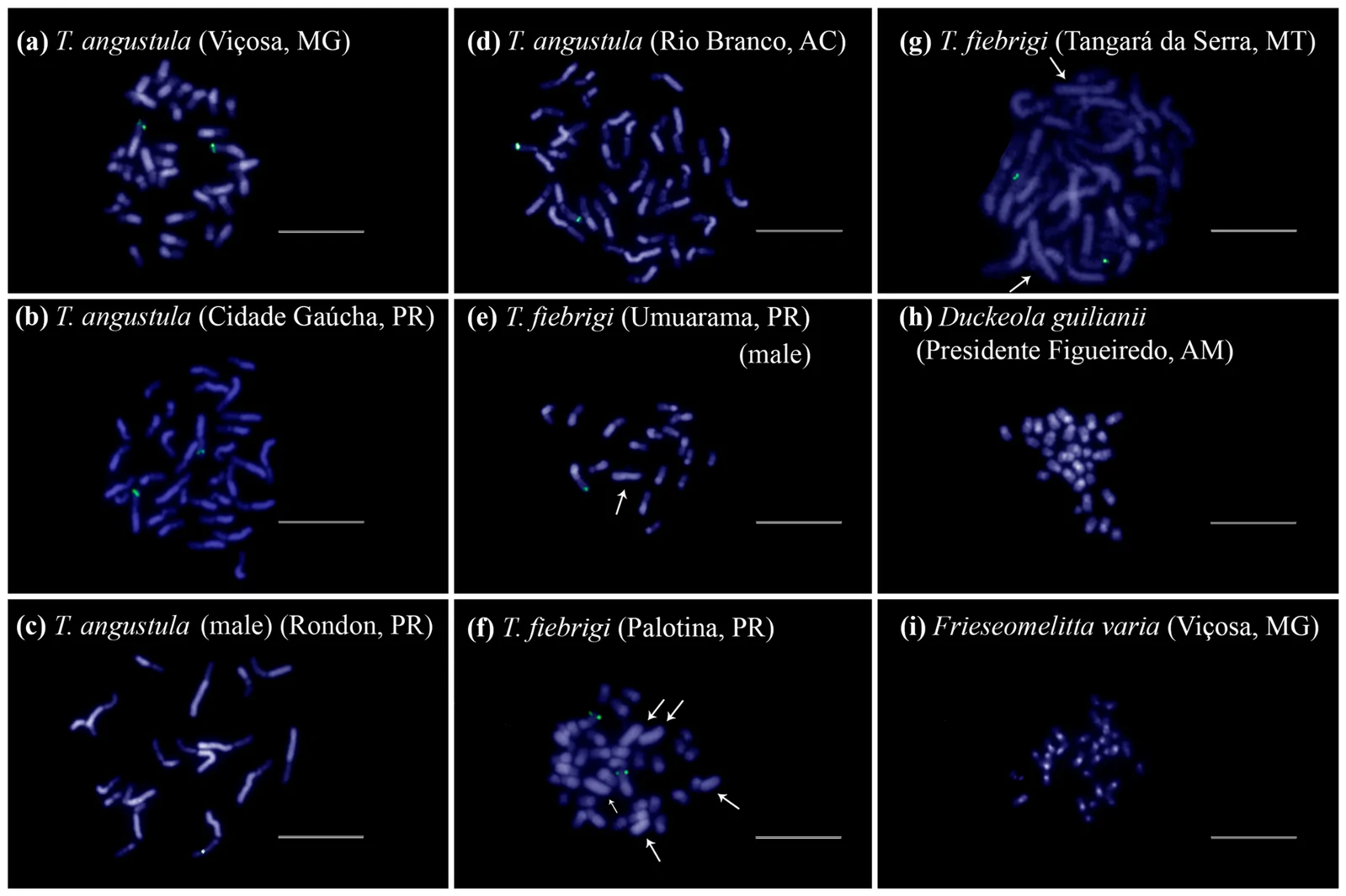
The chromosomal distribution of TangSat04-229 in the karyotypes of *T. angustula* from (**a**) Viçosa, MG; (**b**) Cidade Gaúcha, PR; (**c**) Rondon, PR; and (**d**) Rio Branco, AC, as well as *T. fiebrigi* from (**e**) Umuarama, PR; (**f**) Palotina, PR; and (**g**) Tangará da Serra, MT. Note its absence in (**h**) *D. guilianii* and (**i**) *F. varia.* Chromosomes are stained with DAPI (blue), and the satDNA signals are shown in green. Arrows indicate the B chromosomes. Scale bars = 10 µm.

FISH assays with the TangSat03-129 probe showed markings on five chromosome pairs across all *T. angustula* populations, whereas it showed great variability among *T. fiebrigi* populations, including different numbers of marked B chromosomes (Figure 5). This probe also yielded positive signals in the closely related *D. guilianii* and *F. varia*, with one chromosome pair marked in each species (Figure 6a,b). We subsequently tested more distantly related Meliponini species and detected positive signals in *F. schrottkyi* (one pair), *P. phrynostoma* (one pair), *N. testaceicornis* (one pair), *S. xanthotricha* (five pairs), and *P. helleri* (five pairs) (Figure 6c–g), whereas no signals were observed in the chromosomes of *M. asilvai*, *M. quadrifasciata*, and *L. muelleri*. FISH assays with the TangSat04-229 probe revealed a single euchromatic cluster on one chromosome pair in both *T. angustula* and *T. fiebrigi* populations, whereas no signals were detected in *D. guilianii* or *F. varia* (Figure 7).

**Figure 8:**
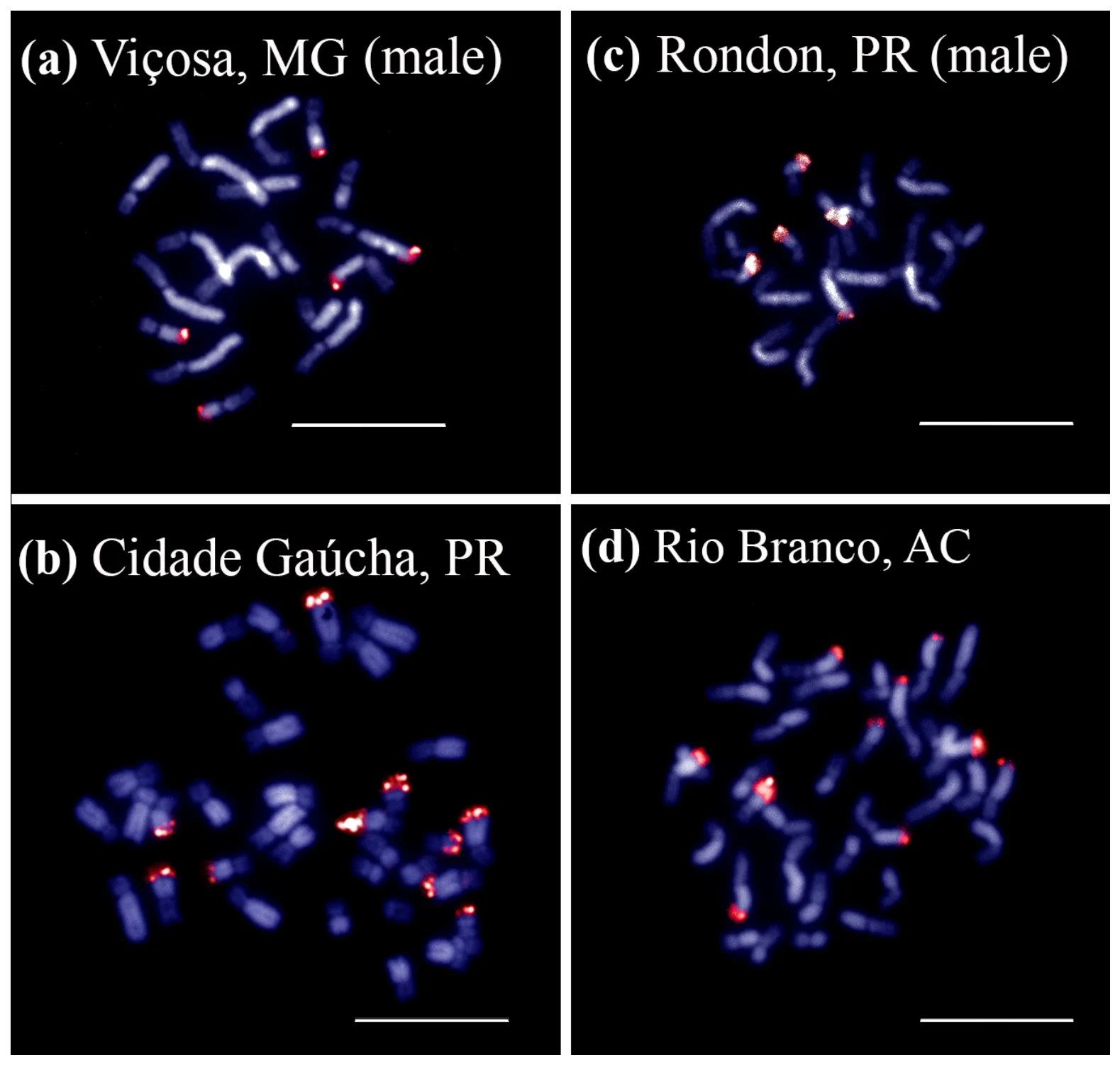
The chromosomal distribution of the 18S rDNA probe in the karyotypes of *T. angustula* from: (**a**) Viçosa, MG; (**b**) Cidade Gaúcha, PR; (**c**) Rondon, PR; and (**d**) Rio Branco, AC. Chromosomes are stained with DAPI (blue), and the rDNA signals are shown in red. Scale bars = 10 µm.

**Figure 9:**
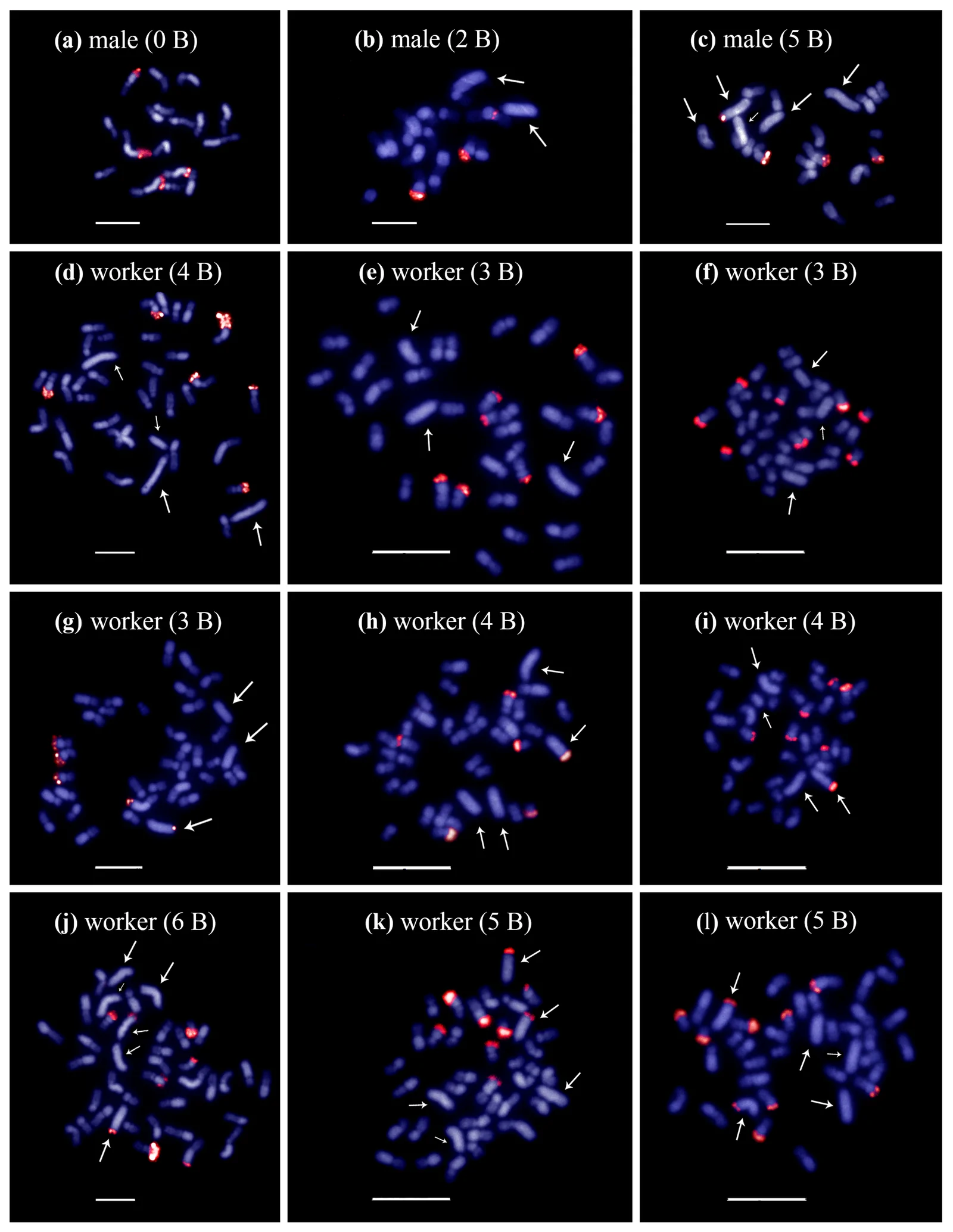
The chromosomal distribution of the 18S rDNA probe in the karyotypes of *T. fiebrigi* males (**a**–**c**) and workers (**d**–**l**). Note the great variability in the number of B chromosomes (arrows). Chromosomes are stained with DAPI (blue), and the rDNA signals are shown in red. Scale bars = 5 µm.

The ribosomal probe revealed the same number of markings in all *T. angustula* populations from Minas Gerais, Paraná, and Acre states: 10 markings on females and 5 on males (Figure 8). On the other hand, a great variability regarding the number of chromosomes marked by the 18S probe was found among *T. fiebrigi* colonies, including different numbers of marked B chromosomes (Figure 9). Among males, we observed three or five markings on the A complement, with some individuals also displaying one marked B chromosome (Figure 9a–c). Among workers, the number of marked B chromosomes varied from 0 to 2. Some individuals showed markings only on the A chromosomes, varying in the range of six, seven, or eight marks (Figure 9d–f). Others had one B chromosome marked plus four, five, or six marked A chromosomes (Figure 9g–i). Finally, some individuals exhibited two marked B chromosomes plus six, seven, or eight marked A chromosomes (Figure 9j–l).

## 4. Discussion

To date, only a handful of stingless bees have had their satDNAs characterized using a cytogenomic approach, namely *M. quadrifasciata*, *M. scutellaris*, *T. hyalinata*, *F. varia*, and *T. angustula* ([8,9,11] and present study). Among these species, *T. angustula* has the fewest number of satDNAs; only four families were detected. The low number of recovered satDNA families may be related to the methodological limitations of the clustering-based approach, which may fail to detect satellite DNAs present at extremely low abundance or exhibiting high sequence divergence [29,30]. Among the recovered satellites, TangSat01-304 and TangSat02-316 had an abundance peak at low divergences (<5%), indicating recent amplifications of these families with high sequence homogenization, whereas the other two satDNA families, TangSat03-129 and TangSat04-229, had an abundance peak at higher divergences (>5%), indicating the accumulation of mutations in these sequences [7].

These four satDNAs accounted for 56.40% of the analyzed fraction of the *T. angustula* genome. On the other hand, the 13 satDNA families found in *Melipona* represent as little as 2.84% of the *M. quadrifasciata* genome and as much as 38.41% of the *M. scutellaris* genome [11]. The seven families found in *F. varia* represent 11.22% of its genome [8], whereas the eight families found in *T. hyalinata* represent 16.56% of its genome [9]. Among these species, *M. quadrifasciata* has the smallest nuclear genome size (1C = 0.27 pg), followed by *F. varia* (1C = 0.47 pg), *T. hyalinata* (1C = 0.53 pg), *T. angustula* (1C = 0.95 pg), and *M. scutellaris* (1C = 1.08 pg) [23,24,28,38]. These results highlight the significant role of satellite DNA in the evolution of the nuclear genome size in stingless bees, indicating that genome size expansion is largely driven by the accumulation of repetitive DNA. In other insect groups, larger genomes have also been positively correlated with higher amounts of repetitive DNA [39,40,41,42,43,44].

Consequently, this may explain the discrepancies between nuclear genome size estimates based on whole-genome sequencing data and those generated by flow cytometry in species with large amounts of repetitive DNA. The estimated genome size of *T. angustula* from the complete genome sequencing data was 1C = 0.29 pg or 284.49 Mb [25]. This amount represents roughly a third of the genome size estimated by flow cytometry, 1C = 0.95 pg or 929.1 Mb [28]. The same pattern is observed in *F. varia*, where the genome size estimated from the complete genome assembly (1C = 0.28 pg or 275 Mb [45]) represents just over half of the estimate obtained by flow cytometry (1C = 0.48 pg or 469.44 Mb [24]). The complete genome sequencing of complex regions, such as repetitive and centromeric regions, becomes increasingly difficult as genome size increases due to the greater abundance of repetitive sequences in heterochromatin [46,47,48].

In contrast, this discrepancy is not observed in species with low proportions of repetitive DNA, such as *M. quadrifasciata*, where the genome size estimated from the complete genome assembly (1C = 0.27 pg or 261 Mb [49]) matches the value obtained by flow cytometry (1C = 0.27 pg or 264 Mb [38]). The same is observed in *Melipona bicolor* Lepeletier, 1836, another species with low amounts of heterochromatin in its karyotype [50], where the genome size estimated from the complete genome assembly (1C = 0.26 pg or 259 Mb [51]) closely matches the estimate obtained by flow cytometry (1C = 0.28 pg or 274 Mb [38]).

There is also a positive correlation between the amount of repetitive DNA in the genome and the proportion of heterochromatin in the chromosomes, with greater proportions of repetitive DNA associated with larger amounts of heterochromatin. The karyotypes of *M. scutellaris* and *T. angustula* are mainly composed of heterochromatin, and the most abundant satDNAs MscuSat01-195 and TangSat01-304, respectively, represent the main constituents of the heterochromatin in the chromosomes of each species ([11] and present study).

Our results demonstrated that TangSat01-304 from *T. angustula* corresponds to the Bsp 68I restriction DNA fragment described by Pereira et al. [15]. This satellite is shared only with the sister species *T. fiebrigi*, including its B chromosomes, being absent from the closely related genera *Duckeola* and *Frieseomelitta*, as well as from other stingless bees ([15] and present study). The sharing of all satDNA families between *T. angustula* and *T. fiebrigi* populations across Brazil, from north to south, indicates a close evolutionary relationship between these two species, which is consistent with their relatively recent divergence approximately 5 Mya [10].

On the other hand, TangSat02-316 is shared with *Duckeola* and *Frieseomelitta*, being present at the centromeres of *Tetragonisca* and *Duckeola* and as part of the main heterochromatic repertoire of *Frieseomelitta*. This satellite belongs to the same satDNA family as FvarSat01-306 from *F. varia* and has also been identified in other Meliponini genera, such as *Friesella*, *Schwarziana*, *Partamona*, *Scaura*, and *Scaptotrigona*, indicating an origin dating back approximately 44 Mya [8]. The same authors that made this identification also showed the loss of this satellite family in *Cephalotrigona*, *Trigona*, *Tetragona*, and *Geotrigona*. Altogether, these results highlight the dynamic evolutionary pathways a satDNA can follow after its emergence, potentially being amplified or lost in different lineages. The loss of this satDNA family in *T. hyalinata* is accompanied by the emergence of new satellites, such as ThyaSat01-301 occupying the whole heterochromatin extension of the chromosomes and ThyaSat02-300 restricted to the centromeric region [9].

The presence of TangSat02-316 in the centromeric regions of all *T. fiebrigi* A chromosomes and in the euchromatic extremity of the B chromosomes may indicate the location of the centromeres in these extra chromosomes (Figure 4). This would mean that all *T. fiebrigi* B chromosomes are morphologically acrocentric. It is uncertain why the centromeres of the B chromosomes are not distinguishable in some species [28]. The presence of repetitive sequences, such as satDNAs and TEs, in the centromeres of different species may be related to the rapid changes in centromere composition and may have an impact on its proper function (reviewed in [4]). The TangSat02-316/FvarSat01-306 family is restricted to the centromeres of *Tetragonisca*, *Duckeola*, and *Scaura* chromosomes and perhaps could have been co-opted to centromeric functions. But, for unknown reasons, this repetitive family grew to become the main heterochromatic component of *Partamona*, *Scaptotrigona*, *Frieseomelitta*, *Friesella*, and *Schwarziana* chromosomes [8], perhaps changing its role to other functions in the genome, such as heterochromatin organization.

TangSat03-129 is also shared between *Tetragonisca* (Figure 5) and other Meliponini genera (Figure 6). Neotropical Meliponini species are phylogenetically subdivided into three clades [10,14], clade 1 represented here by *Leurotrigona*, clade 2 represented by *Melipona* species, and the remaining studied species belonging to clade 3. In this context, TangSat03-129 is shared only among species from clade 3, indicating that its origin dates back at least 44 Mya, similarly to the TangSat02-316/FvarSat01-306 family.

Interestingly, TangSat03-129 colocalizes with rDNA genes and has spread across the karyotypes of species harboring multiple rDNA clusters. It is hypothesized that a single pair of 18S rDNA represents the plesiomorphic state in Neotropical Meliponini [19]. Accordingly, a single pair of 18S rDNA and a single TangSat03-129 locus are observed in many of the analyzed species, i.e., *Duckeola*, *Frieseomelitta*, *Friesella*, *Plebeia*, and *Nannotrigona*, whereas the number of these loci has expanded in *Tetragonisca*, *Scaptotrigona*, and *Partamona* ([19,52] and present study). The colocalization of TangSat03-129 with rDNA clusters in both basal and derived species within Meliponini species from clade 3 may suggest an evolutionary association that originated at least 44 Mya and has been maintained throughout the diversification of this clade. Therefore, the insertion of this satDNA between ribosomal genes resulted in the satellite sequence spreading as 18S expanded to additional chromosomes in certain species.

We observed a conserved number of 18S signals across all *T. angustula* populations, from northern (Acre) to southern Brazil (Paraná). Contrary to this conservatism, *T. fiebrigi* presents a great variability in the number of marked chromosomes, including markings on the B chromosomes. The Bs probably originated from chromosome breaks in the heterochromatic arms of the A complement of *T. fiebrigi* (intraspecific origin) [28]. As some breaks likely occurred in chromosomes carrying ribosomal and TangSat03-129 clusters, 18S rDNA/TangSat03-129 were incorporated into some B chromosomes, resulting in an uneven number of signals in the A complement of *T. fiebrigi*. This uneven distribution resulted in extensive variation in subsequent generations, as observed in both males and workers (Figure 5 and Figure 9).

The last satellite family TangSat04-229 was restricted to the genus *Tetragonisca* and formed a single cluster in the euchromatic arm of one chromosome pair in both *T. angustula* and *T. fiebrigi*. Although satDNAs are described as major constituents of heterochromatin, their presence in the euchromatic region has been described [32,53,54,55,56]. To date, not all satDNAs identified in Meliponini genomes have been mapped to the chromosomes of the species in which they were identified. Therefore, future studies investigating the chromosomal distribution of different satDNA families will reveal whether satDNAs in euchromatic regions are exclusively characteristic to *Tetragonisca* or are widespread among stingless bees.

Here, we reported unique and shared satDNA families within *Tetragonisca* and other stingless bees. The presence of shared sequences over long evolutionary periods is consistent with the library hypothesis, which predicts a common set of families inherited from a common ancestor, which can be independently amplified or lost [57,58]. On the other hand, the presence of unique sequences highlights the importance of satellite divergence and diversification in reproductive isolation, contributing to the speciation process (reviewed in [59]).

Besides satDNAs, TEs are also known to be related to genome size expansion in many species [11,42,43,49,60,61]. Given the large genomes observed in *Tetragonisca* species [28], the presence of these elements in their genomes is also expected. Investigating this possibility would be an interesting avenue for future research.

## Figures and Tables

**Table 1:** Stingless bee sampling locations across different Brazilian regions. Diploid number (2*n*) and presence of B chromosomes are also shown.

Species	Sampling Location	2*n*
*T. angustula*	Viçosa, Minas Gerais	34
*T. angustula*	Cidade Gaúcha, Paraná	34
*T. angustula*	Rondon, Paraná	34
*T. angustula*	Rio Branco, Acre	34
*T. fiebrigi*	Tapejara, Paraná	34 + 1B
*T. fiebrigi*	Umuarama, Paraná	34 + 1B up to 3B
*T. fiebrigi*	Goioerê, Paraná	34 + 1B up to 4B
*T. fiebrigi*	Palotina, Paraná	34 + 1B up to 7B
*T. fiebrigi*	Tangará da Serra, Mato Grosso	34 + 1B up to 3B
*D. guilianii*	Presidente Figueiredo, Amazonas	30
*F. varia*	Uberlândia, Minas Gerais	30
*Friesella schrottkyi*	Viçosa, Minas Gerais	34
*Plebeia phrynostoma*	Espírito Santo	34
*Nannotrigona testaceicornis*	Januária, Minas Gerais	34
*Scaptotrigona xanthotricha*	Viçosa, Minas Gerais	34
*Partamona helleri*	Viçosa, Minas Gerais	34
*Melipona asilvai*	Ribeirão Preto, São Paulo	18
*Melipona quadrifasciata*	Viçosa, Minas Gerais	18
*Leurotrigona muelleri*	Ribeirão Preto, São Paulo	16

## Data Availability

The sequences of the satellite DNA families were deposited in the GenBank/NCBI DNA databases under accession numbers PZ803731 to PZ803734.

## Supplementary Materials

The following supporting information can be downloaded at https://www.mdpi.com/article/10.3390/genes17101254/s1, Figure S1: Geographical sampling locations of *T. angustula* (black circles): 1. Rio Branco, AC, 2. Viçosa, MG, 3. Cidade Gaúcha, PR, 4. Rondon, PR; and *T. fiebrigi* (yellow circles): 5. Tapejara, PR, 6. Goioerê, PR, 7. Palotina, PR, 8. Umuarama, PR, 9. Tangará da Serra, MT; Figure S2: Alignment of the most abundant satellite DNA family in the *T. angustula* genome, TangSat01-304, with the restriction enzyme Bsp 68I recognition site, showing its location at positions 291–296 of the 304-bp satellite monomer; Figure S3: Comparison of TangSat02-316 from the *T. angustula* genome and FvarSat01-306 from the *F. varia* genome revealed 80.3% sequence identity, indicating that these sequences belong to the same satellite DNA family.
