# Supplementary material for: Cytogenomics of Tetragonisca angustula Reveal Unique and Shared satDNA Families Within Tetragonisca and Closely Related Genera (Apidae: Meliponini)
Source: Genes (Basel). 2026 Oct 10;17(10):1254. doi: 10.3390/genes17101254 (PMC13704234; doi:10.3390/genes17101254)
Supplement: Supplementary file 1 [file genes-17-01254-s001.zip › genes-4590337-supplementary.pdf]

Supplementary Materials

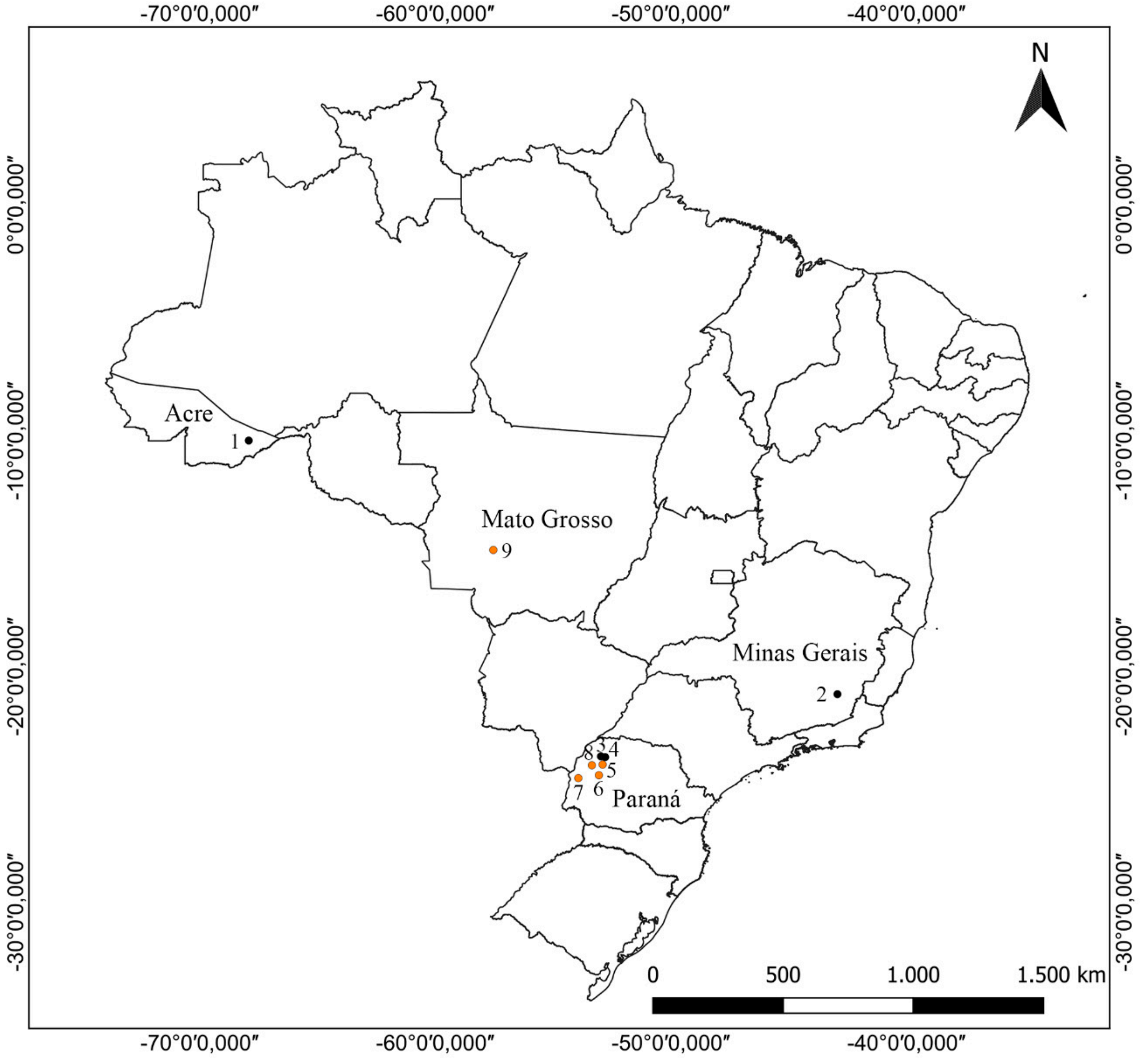

Figure S1. Geographical sampling locations of *T. angustula* (black circles): 1. Rio Branco, AC, 2. Viçosa, MG, 3. Cidade Gaúcha, PR, 4. Rondon, PR; and *T. fiebrigi* (yellow circles): 5. Tapejara, PR, 6. Goioerê, PR, 7. Palotina, PR, 8. Umuarama, PR, 9. Tangará da Serra, MT.

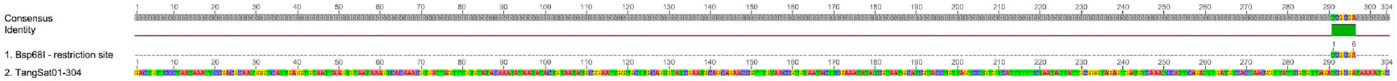

Figure S2. Alignment of the most abundant satellite DNA family in the *T. angustula* genome, TangSat01-304, with the restriction enzyme Bsp 68I recognition site, showing its location at positions 291–296 of the 304-bp satellite monomer.

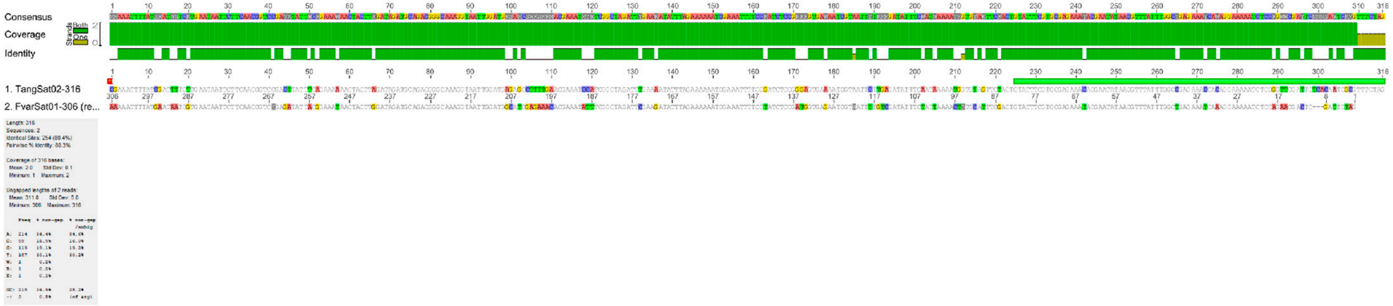

Figure S3. Comparison of TangSat02-316 from the *T. angustula* genome and FvarSat01-306 from the *F. varia* genome revealed 80.3% sequence identity, indicating that these sequences belong to the same satellite DNA family.
